# *Cryptococcus neoformans* var. *gattii* isolate with Unusual Morphology

**DOI:** 10.1590/0037-8682-0412-2019

**Published:** 2020-01-27

**Authors:** Anil Kumar, Malini Eapen, Rosamma Philip

**Affiliations:** 1 Department of Microbiology, Amrita Institute of Medical Sciences, Amrita Vishwa Vidyapeetham, Ponekara, Kochi, Kerala India.; 2 Department of Pathology, Amrita Institute of Medical Sciences, Amrita Vishwa Vidyapeetham, Ponekara, Kochi, Kerala India.; 3 Department of Marine Biology, Microbiology and Biochemistry, School of Marine Sciences, Cochin University of Science and Technology, Fine Arts Avenue, Kochi, Kerala, India.

An immunocompetent 24-year-old woman was referred to our hospital with viral meningitis that did not respond to treatment. Brain magnetic resonance imaging (MRI) revealed signs suggestive of acute disseminated encephalomyelitis. Cerebrospinal fluid (CSF) showed glucose levels of 64.8 mg/dL and protein levels of 65.6 mg/dL. CSF microscopy showed 42 cells/mm^3^, including polymorphs, lymphocytes, and yeast cells with atypical morphology. Grocott-Gomori's-methenamine-silver staining, India ink staining, and calcofluor white staining of CSF demonstrated the existence of unusual morphological features like pseudohypha formation, chains of budding yeasts, and structures resembling germ tubes (Figures A, Figure B and Figure C). Most of the cells were encapsulated. Sequencing identified yeast isolates grown on blood and CSF cultures as *Cryptococcus neoformans*var*. gattii*. The isolate was sensitive to voriconazole, amphotericin B, fluconazole, and flucytosine. The patient succumbed to the infection despite initiating treatment with liposomal amphotericin B.

**FIGURE A:**
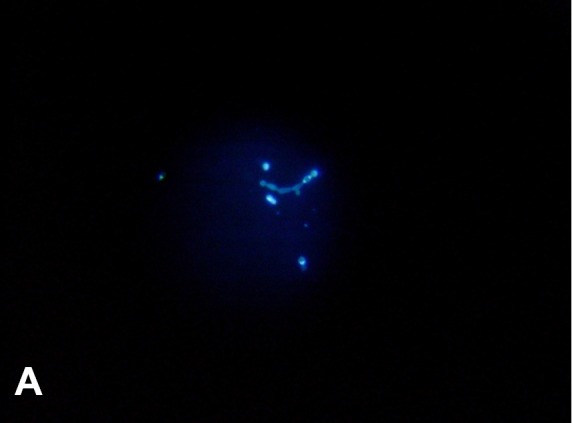
Calcofluor white staining of CSF showing pseudohyphal forms (40X).

**FIGURE B:**
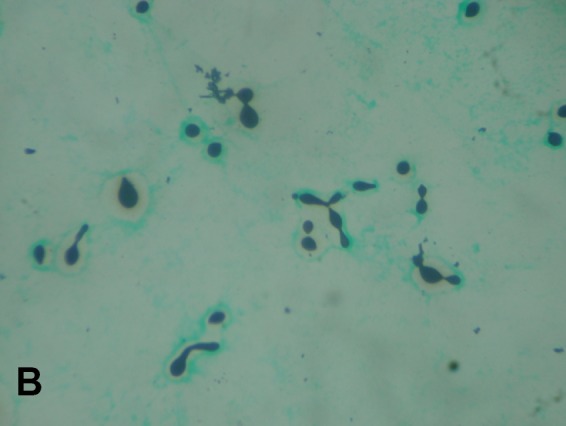
Gomori-methenamine-silver staining showing encapsulated pseudohyphal forms and germ-tube formation (100X).

**FIGURE C:**
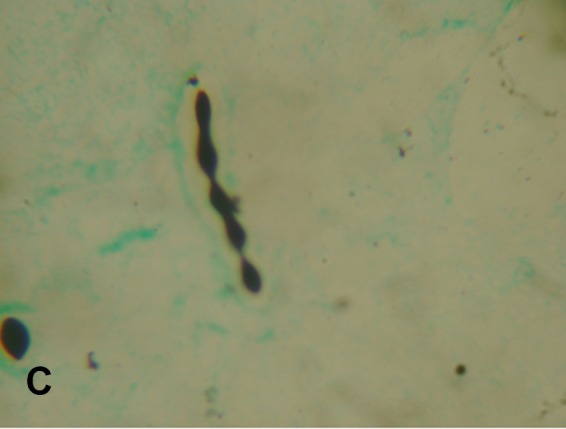
Gomori-methenamine-silver staining showing encapsulated chains of budding yeasts (100X).

*Cryptococcus* can deviate from its characteristic morphology as encapsulated budding yeast cells, presenting as pseudohyphae or germ tube-like structures^1^. In such instances, cryptococcal antigen detection using latex agglutination or culture identification should be used for a definitive diagnosis.
